# Do Chinese Readers Follow the National Standard Rules for Word Segmentation during Reading?

**DOI:** 10.1371/journal.pone.0055440

**Published:** 2013-02-08

**Authors:** Ping-Ping Liu, Wei-Jun Li, Nan Lin, Xing-Shan Li

**Affiliations:** 1 Key Laboratory of Behavioral Science, Institute of Psychology, Chinese Academy of Sciences, Beijing, China; 2 Graduate University of Chinese Academy of Sciences, Beijing, China; New York University, United States of America

## Abstract

We conducted a preliminary study to examine whether Chinese readers’ spontaneous word segmentation processing is consistent with the national standard rules of word segmentation based on the *Contemporary Chinese language word segmentation specification for information processing* (*CCLWSSIP*). Participants were asked to segment Chinese sentences into individual words according to their prior knowledge of words. The results showed that Chinese readers did not follow the segmentation rules of the *CCLWSSIP*, and their word segmentation processing was influenced by the syntactic categories of consecutive words. In many cases, the participants did not consider the auxiliary words, adverbs, adjectives, nouns, verbs, numerals and quantifiers as single word units. Generally, Chinese readers tended to combine function words with content words to form single word units, indicating they were inclined to chunk single words into large information units during word segmentation. Additionally, the “overextension of monosyllable words” hypothesis was tested and it might need to be corrected to some degree, implying that word length have an implicit influence on Chinese readers’ segmentation processing. Implications of these results for models of word recognition and eye movement control are discussed.

## Introduction

Words are generally considered to be the basic meaningful unit of language. Most printed alphabetic writing systems provide readers with unambiguous markers that segment sentences into individual words, such as interword spaces in English. If a group of readers is given an English sentence and asked to count the number of words in the sentence, the answer must be definite in most cases. For some ideographic scripts, such as Chinese, there are no explicit cues to tell readers where a word begins or ends in a serial string of characters. If a group of native Chinese speakers are asked to count the number of words in a Chinese sentence, the answers must be diverse. Although some studies have mentioned that Chinese readers often disagree on the word boundaries for the same text [1]–[8], few psycholinguistic studies further investigated the mechanism of inconsistent word segmentation during Chinese reading. Additionally, although there are several computational models of word recognition and eye movement control in Chinese, few models take ambiguous word segmentation into account in the context of sentence reading [8]–[11]. Thus, understanding how readers break the continuous string of characters into individual words remains one of the fundamental issues in word recognition and language comprehension in Chinese.

It is known that most Chinese readers experience difficulty in agreeing on word boundaries in a serial string of characters. This fact raises a critical question of whether Chinese words have psychological reality for Chinese readers. A number of studies have suggested that words have psychological reality for Chinese readers. First, Bai et al. (2008) found that inserting spaces after each character inhibited sentence reading, while adding spaces between words did not. This study clearly indicated that word units rather than individual characters play an important role in Chinese reading. Second, several studies have reported that individual Chinese characters can be detected more efficiently in a word than in a string of characters that does not constitute a word [8], [12]–[14], suggesting that the word contexts facilitate character perception. Third, some studies have demonstrated that the word properties (e.g., word frequency, word predictability) have stronger effects than character properties on fixation durations and word skipping in Chinese [6], [15], [16]. These findings all demonstrated that words do have psychological reality for Chinese readers.

Although Chinese word segmentation has not attracted much attention in psycholinguistic studies, it has been studied for many years in computational linguistics [17]–[24]. In this research field, most researchers are more concerned with automatic word segmentation techniques, which play an important role in information processing, such as in automatic speech recognition systems, information retrieval, machine translation, human-robot interaction, and so on. In the last decade, several pragmatic approaches for automatic Chinese word segmentation have been proposed. The precision and recall rates for segmentation can be above 90% [20], [25]. Automatic word segmentation techniques have improved and have been well defined as “segmentation specification+lexicon+segmented corpus” [19]. Notably, word segmentation specification (e.g., dictionary-based or statistically-based) is the fundamental question in automatic Chinese word segmentation.

Word segmentation specification plays a critical role in automatic Chinese word segmentation techniques. One of the most influential word segmentation specifications is the *Contemporary Chinese language word segmentation specification for information processing* (*CCLWSSIP*), which has been authorized as the national standard for the rules of word segmentation units in Chinese reading [26]. According to the *CCLWSSIP*, each syntactic category (i.e., the adjectives, nouns, numeral, quantifiers, verbs, adverbs, prepositions, conjunctions, auxiliary words, etc.) can be considered as a segmented word unit (SWU). Generally, the linguistic criteria of the *CCLWSSIP* were established to avoid word boundary ambiguity for automatic Chinese word segmentation. However, it is unknown whether ordinary native Chinese readers follow the word segmentation rules of the *CCLWSSIP.* Thus, one purpose of the present study is to test whether word segmentation rules used by ordinary Chinese readers are consistent with the rules of the *CCLWSSIP.*

For the issues of word segmentation rules used by ordinary Chinese readers, there are three possibilities. First, Chinese readers may reach a consensus on word boundaries, and their spontaneous word segmentation processing may follow the rules of the *CCLWSSIP*. Second, Chinese readers may reach a consensus on word boundaries, but their segmentation processing may not follow the rules of the *CCLWSSIP*. Third, Chinese readers may not reach a consensus on the word boundaries for the same text, thus their segmentation processing rules could be inconsistent with the rules of the *CCLWSSIP*. The present study attempted to test the three possibilities, and explored whether ordinary Chinese readers followed the rules of the *CCLWSSIP* when they were asked to segment Chinese sentences into individual words.

According to previous research, it seemed that Chinese readers may not follow the rules of the *CCLWSSIP* to identify individual words in a serial string of characters. Peng and Chen (2004) reported a phenomenon that most Chinese readers tended to combine monosyllables with disyllables to form a “word”. They named the tendency as “overextension of monosyllable words”, which may lead to the word segmentation inconsistency. The finding suggests that word length may have an effect on word segmentation processing of Chinese readers. If the word defined by the *CCLWSSIP* contains 1 character, Chinese readers may tend to combine the 1-character word with other characters to form a single word. For instance, the string of characters “
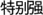
” (very strong) is more likely to be considered as a single word by most ordinary Chinese readers rather than two words “
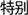
” (i.e., it is a disyllable and can be translated as “very”) and “
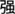
” (i.e., it is a monosyllable and can be translated as “strong”) based on the rules of the *CCLWSSIP*. Thus, we hypothesized that Chinese readers may not follow the rules of the *CCLWSSIP* in some cases.

Furthermore, there is little psycholinguistic research to clarify the cases in which consistent or inconsistent word segmentation occurs for ordinary readers in Chinese reading. One of the limited studies that directly examined the inconsistent word segmentation during Chinese reading was reported by Hoosain (1992). In the study, fourteen undergraduates were invited to mark word boundaries from two sets of materials that consisted of a paragraph and nine sentences. Specifically, the participants in the study were native Cantonese speakers in Hong Kong, while their medium of instruction for most school courses was English. The results showed substantial disagreement regarding the word boundaries in the limited materials. Given that Hoosain’s (1992) results were very brief, it is important to stimulate further research on word segmentation and recognition in Chinese. In the past 20 years, education in China has rapidly developed and the Chinese textbooks have substantially changed. Perhaps some of the findings observed in last century need to be tested in modern times.

The purpose of the present study was to explore whether the segmentation rules of Chinese readers were consistent with the rules of the *CCLWSSIP*. Following Hoosain’s (1992) research method, we examined the rules of word segmentation by native Chinese readers who were invited to segment Chinese sentences into individual words. Two hundred sentences were selected as the test materials, and one hundred and forty two native Chinese speakers were recruited to participate in the investigation. Because the word syntactic categories were used to set the standard rules of word segmentation by the *CCLWSSIP*, they were also used as variables to examine the segmentation rules of Chinese readers in the present study. Each segmented word unit was considered as a target word. If the Chinese readers did not follow the segmentation rules of the *CCLWSSIP*, we would analyze the syntactic categories of the adjacent words, since the sentence context and syntactic relationship between these consecutive words may play some roles in Chinese word segmentation [8], [27]. The results of this study may shed light on word segmentation issues in Chinese reading.

## Methods

### Ethics Statement

The study was approved by the Institutional Review Board of the Institute of Psychology, Chinese Academy of Sciences. All participants provided written, informed consent before taking part in our experiments.

### Participants

One hundred and forty two undergraduates or graduate students at universities in Beijing near the Institute of Psychology were paid to take part in the experiment. All of them were native Chinese speakers and had either normal or corrected-to-normal vision.

### Apparatus

The materials were presented on a 19-inch LCD monitor with a resolution of 1,440×900 pixels and a refresh rate of 60 Hz.

### Materials

Two hundred sentences were obtained from an online corpus (Center for Chinese Linguistics PKU, http://ccl.pku.edu.cn:8080/ccl_corpus/index.jsp?dir=xiandai). Some of these sentences were slightly edited to prevent semantic ambiguities. The sentences were all between 20 and 38 characters in length (*M* = 29.5, *SD* = 3.8).

### Task and Procedure

The instruction and materials were presented on a computer monitor and were divided into two parts. The first part was to ask participants to write down the concept of Chinese word based on their prior knowledge. In the second part, the participants were asked to segment the normal Chinese sentences into individual words by slashes (“/”). Thirty-two participants were asked to segment one hundred sentences, which took approximately 50–60 min. Because the experiment was lengthy, some of the participants may feel exhausted to complete the segmentation task. Thus, another one hundred and ten participants were asked to segment fifty sentences, which took approximately 30 min. Overall, each sentence was segmented at least by forty participants.

### Data Analyses

We analyzed the data based on the traditional syntactic categories, including function words and content words. The adverbs, auxiliary words, conjunctions, exclamations, and prepositions were considered to be function words based on the dictionary of function words [28]–[30]. The adjectives, nouns, numerals, quantifier, pronouns, and verbs were regarded as content words [2], [31]. We reported the level of agreement on word boundaries for the function words and content words. The illustration of SWU and the coding of the agreement for word boundaries near the SWU are presented in the Appendix S1.

The experimental sentences contained 5,588 characters and 312 punctuations. In total, there were 3,388 SWU that were followed by a word boundary according to participants’ segmentation decisions. The concept of SWU was delimited as the minimum word unit based on the word segmentation results by participants and *Chinese Lexicon* (2003). We did not analyze the SWU at the beginning of sentences because the beginning of the sentences can mark the left boundary of the SWU. Moreover, SWU near the punctuations were excluded from the analysis, because punctuations can also mark the left or right word boundaries of SWU. Finally, we analyzed 2,724 SWU for which the possible word boundaries were marked.

The syntactic categories of all of the SWU were marked according to part-of-speech tagging by *ICTCLAS* (Institute of Computing Technology, Chinese Lexical Analysis System, http://ictclas.org/) [32], *Modern Chinese Dictionary* (2005), and *Function words in Modern Chinese Dictionary* [28], [30]. Meanwhile, three native Chinese speakers were invited to evaluate the syntactic categories based on the dictionary and their prior knowledge, and the agreement rate for the 2,724 SWU was 96%. In total, there were 1,001 SWU of function words and 1,723 SWU of content words. The average agreement proportions for word boundary decisions were clear in that they revealed the cases in which the consistent and inconsistent word segmentation occurred. In addition, single-sample *t* tests were performed to compare whether the agreement proportion for word boundaries against a value of .50. If the average agreement proportion was significantly higher than .50, it indicated that a majority of the participants agreed that there was word boundary at that position. If the proportion was significantly lower than .50, it indicated that a majority of participants disagreed that there was a word boundary at that position.

## Results and Discussion

### Global Analyses

**Figure 1:**
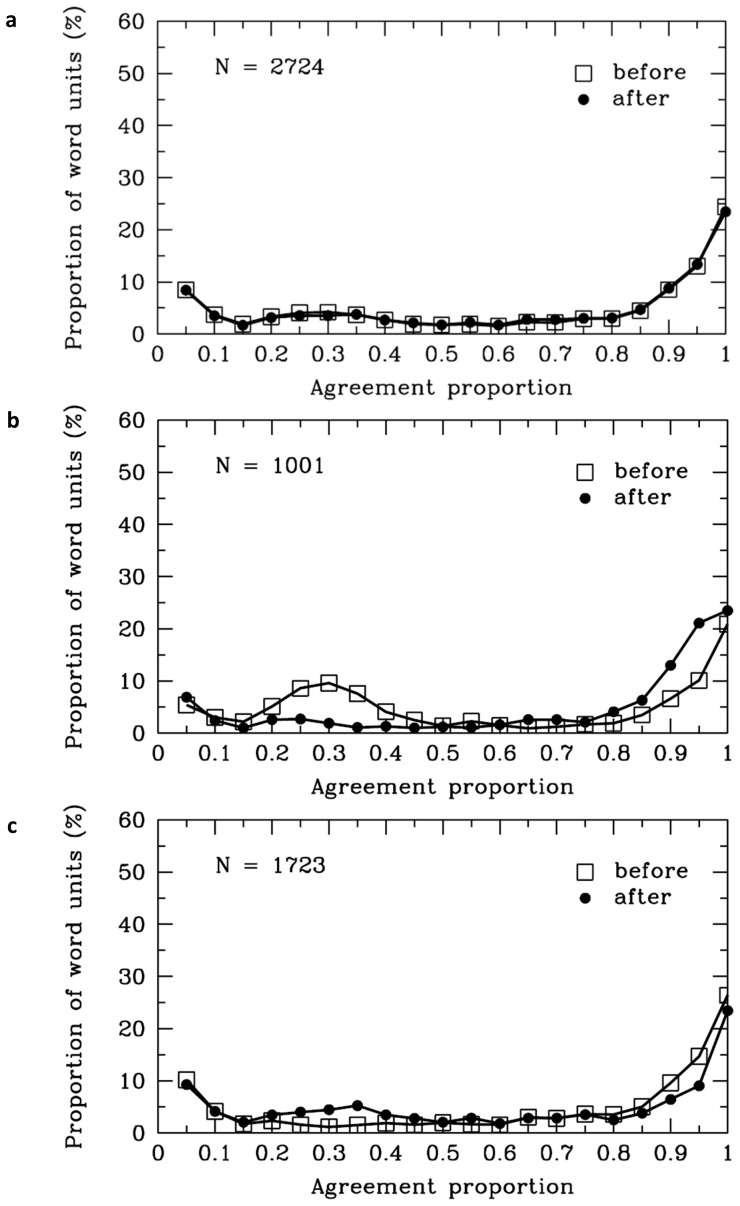
Global analyses. Frequency distribution of agreement proportions for word boundaries before (open squares) and after (filled circles) the SWU (a: total SWU; b: total SWU of function words; c: total SWU of content words). The y-axis represents the proportion of total SWU in the classes. The number of SWU is also displayed in each panel of the figure.

Table 1 shows the average agreement proportions for the decisions that there were word boundaries before and after the SWU of each syntactic category, respectively. The frequency distributions of the agreement proportions are presented in Figure 1a (total words), Figure 1b (total function words), and Figure 1c (total content words). The horizontal axis in this plot represents the average agreement proportions of all the participants for the decisions that there were word boundaries before or after the SWU. The vertical axis represents the proportions of SWU, and the number of SWU is also present in the figure (e.g., N = 2,724 in Figure 1a). The average agreement proportion of all the participants for the decisions that there were word boundaries after the total SWU was .64. The single-sample *t* test showed that the agreement proportions for word boundaries before and after the SWU of function words were significantly higher than .50, all *ps* <.001 (see Table 1). Furthermore, the average agreement proportions results indicated that the participants were more likely to agree there was a boundary after the SWU of function words (*M* = .73, *SD* = .32) than before the SWU of function words (*M* = .57, *SD* = .35), *t* (1000) = 9.17, *p*<.001. In contrast, the participants were more likely to agree there was a boundary before (*M* = .69, *SD* = .35) the content words than after the SWU of content words (*M* = .59, *SD* = .35), *t* (1722) = 8.02, *p*<.001.

**Table 1:** Single Sample t-test Analyses and Agreement Proportion for Word Boundary Before and After the Various Words Units.

	Function words					Content words								Total words
SC	adv.	aux.	conj.	prep.	Total^*^	adj.	n.	num.	qua.	N&Q	pron.	v.	Total^#^	
N	320	471	66	144	1001	231	766	50	104	15	129	428	1723	2724
Boundary before the words														
mean	.79	.31	.94	.74	.57	.83	.67	.69	.08	.54	.82	.78	.69	.65
*SD*	.30	.19	.06	.29	.35	.24	.33	.33	.09	.35	.26	.3	.34	.35
*t*	17.28	−21.41	58.76	9.95	6.38	20.48	14.32	4.12	−45.76	.46	13.81	19.27	23.47	22.23
*p*	<.001	<.001	<.001	<.001	<.001	<.001	<.001	<.001	<.001	.65	<.001	<.001	<.001	<.001
Boundary after the words														
mean	.48	.88	.80	.74	.73	.34	.66	.18	.76	.68	.52	.62	.59	.64
*SD*	.36	.19	.21	.24	.32	.21	.33	.28	.24	.35	.37	.37	.35	.35
*t*	−.76	43.29	11.18	11.98	23.04	−11.53	13.43	−8.04	11.21	1.95	.72	6.98	10.68	21.44
*p*	.45	<.001	<.001	<.001	<.001	<.001	<.001	<.001	<.001	.07	.48	<.001	<.001	<.001

Note. Probabilities are in percentages. SC = syntactic categories; mean = mean agreement proportion; SD = standard deviations;

adv. = adverbs; aux. = auxiliary words; conj. = conjunctions; prep. = prepositions; total* = total function words; adj. = adjectives;

n. = nouns; num. = numerals; qua. = quantifiers; N&Q = numerals and quantifiers; pron. = pronouns;

v. = verbs; total^#^ = total content words; Total = all the word units.

As shown in Table 1, the patterns of distribution frequency of agreement proportions varied across the SWU of different syntactic categories. There are many factors such as lexical knowledge, syntactical and context information that may affect Chinese readers’ word segmentation processing. To clarify the complex factors, we reported the frequency distribution of the agreement proportion for the SWU of major syntactic categories that were often present in the materials. The results indicated that whether ordinary Chinese readers followed the rules of the *CCLWSSIP*.

### Function Words

#### Auxiliary words

**Figure 2:**
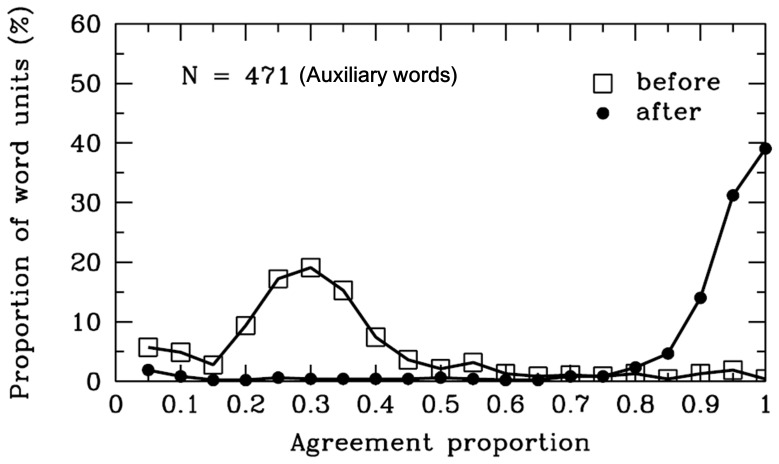
Auxiliary words. Frequency distribution of agreement proportions for word boundaries before (open squares) and after (filled circles) the SWU of total auxiliary words. The x-axis represents the average proportions of all of the participants who indicated that there were word boundaries before or after the SWU. The y-axis represents the proportion of SWU in the classes. The number of SWU is also represented in the figure.

As shown in Table 1, the agreement proportions for word boundaries before (*M* = .31, *SD* = .19) the auxiliary words were significantly lower than .50, *t* (470) = −21.41, *p*<.001. The agreement proportions for word boundaries after (*M* = .88, *SD* = .19) the auxiliary words were significantly higher than .50, *t* (470) = 43.29, *p*<.001. Figure 2 displays the tendency. Among the materials, there were 17 different SWU of the auxiliary words. Of the 471 auxiliary words, 99% were 1-character words such that the participants tended to combine the auxiliary word with the adjacent characters to form a single word. This finding appears to support the “overextension of monosyllable words” hypothesis. One limitation of our materials was that it did not contain all the auxiliary words with equal weight. Approximately 58% of the auxiliary words were “
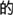
” (i.e., of, in, on, etc.), which has the highest frequency of all Chinese words according to *Chinese Lexicon* (2003). Thus, the results may reflect readers’ segmentation processing of main structural auxiliary words, such as “
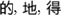
” (i.e., of, in, on, etc.). The results showed that participants tended to mark word boundaries after the auxiliary words, and no word boundaries before the auxiliary words in most cases.

**Figure 3:**
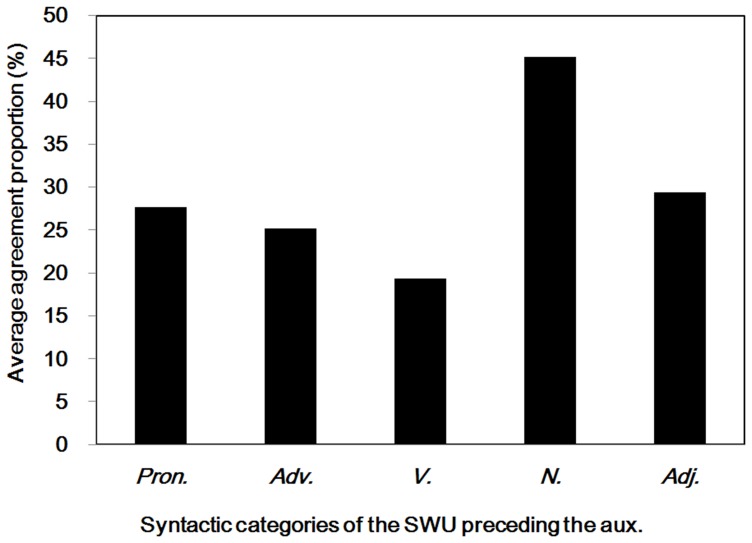
Preceding words and the auxiliary words. Average agreement proportions for word boundaries between the different preceding syntactic categories of SWU and the auxiliary words. Pron. = pronouns; Adv. = adverbs; V. = verbs; N. = nouns; Adj. = adjectives; Aux. = auxiliary words.

Because most participants denied there were word boundaries before the auxiliary words, we analyzed the syntactic categories of SWU preceding the SWU of the auxiliary words. As shown in Figure 3, most of the auxiliary words were preceded by the pronouns, adverbs, verbs, nouns, and adjectives. However, the average agreement proportions between each syntactic category of preceding word units and the auxiliary words were below .50. The results may relate to the properties of auxiliary words, which are often used to supplement other words or to end a sentence; they cannot be used independently. Additionally, the SWU of auxiliary words cannot have a substantial meaning [28], [29]. Thus, the results suggested that few Chinese readers considered the auxiliary word as a single word unit, a finding that was inconsistent with the rules of the *CCLWSSIP*.

#### Adverbs

**Figure 4:**
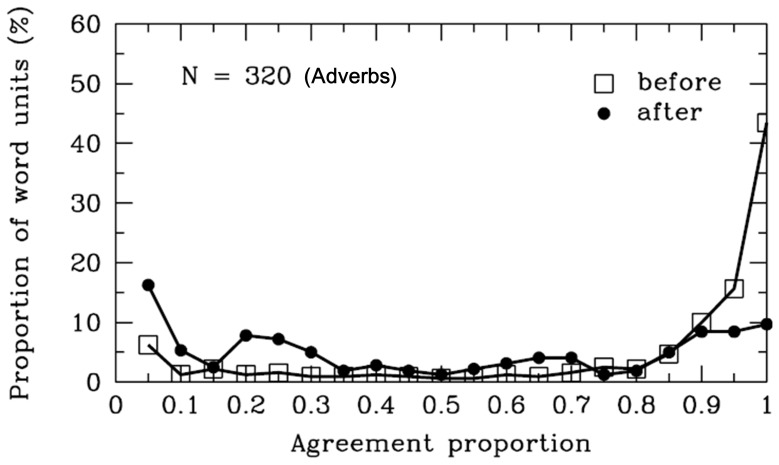
Adverbs. Frequency distribution of agreement proportions for word boundaries before (open squares) and after (filled circles) the adverbs. The y-axis represents the proportion of SWU. The number of SWU is also present in the figure.

**Figure 5:**
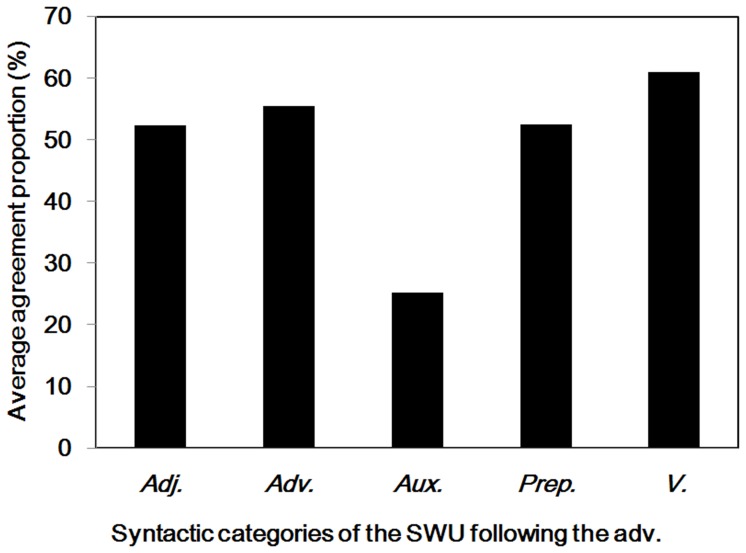
Adverbs and the subsequent words. Average agreement proportions for word boundaries between the adverbs and different subsequent syntactic categories of SWU. Adj. = adjectives; Adv. = adverbs; Aux. = auxiliary words; Prep. = prepositions; V. = verbs.

As shown in Table 1 and Figure 4, the agreement proportion for word boundaries before (*M* = .79, *SD* = .30) the adverbs was significantly higher than .50, *t* (319) = 17.28, *p*<.001. However, the agreement proportion for word boundaries after (*M* = .48, *SD* = .36) the adverbs did not differ significantly from .50, *t* (319) = −.76, *p* = .45. There were 119 different adverbs segmented in the materials, and the word length varied from one to four characters. To examine why most participants did not reach a consensus on the word boundaries after the adverbs, we analyzed the syntactic categories of SWU after the SWU of the adverbs in detail. As shown in Figure 5, most adverbs were followed by the verbs, other adverbs, auxiliary words, adjectives, and others. The average agreement proportions for word boundaries between the adverbs and these different subsequent words were no more than .61.

The results suggested that most Chinese readers were uncertain whether word boundaries exist after the adverbs. The inconsistent word segmentation may relate to the feature of the adverbs, which are used to modify the subsequent verbs, adjectives and other adverbs. The adverbs can be used to show time, scope, degree, modal manner, frequency or negative [28], [29]. In special cases, there are auxiliary words (“
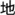
”, which means “of, in, at, etc.”) between the adverbs and the modified ingredients (e.g., “
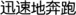
”, the auxiliary word “
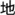
” was inserted between the adverb “
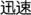
” and the verb “
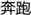
”, which means *run quickly*). Readers tended to combine the adverb and the auxiliary word as a single word. Thus, unlike the rules of the *CCLWSSIP*, the results suggested that Chinese readers disagreed that the adverb could be considered a single word unit.

#### Conjunctions

**Figure 6:**
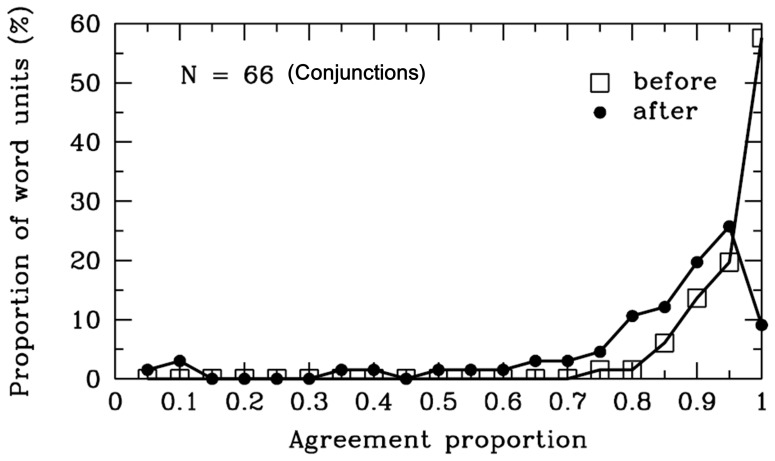
Conjunctions. Frequency distribution of agreement proportions for word boundaries before (open squares) and after (filled circles) the conjunctions. The y-axis represents the proportion of SWU. The number of SWU is also represented in the figure.

As is evident in Table 1 and Figure 6, the agreement proportions for word boundaries before [*M* = .94, *SD* = .06, *t* (65) = 58.76, *p*<.001] and after [*M* = .80, *SD* = .21, *t* (65) = 11.18, *p*<.001] the conjunctions were significantly higher than .50. There were 10 different conjunctions segmented in the materials, and 94% of the 66 conjunctions were 1-character words (i.e., monosyllable words). Interestingly, most participants did not tend to combine the monosyllable conjunction and the adjacent characters to form a single word, and the finding did not support the “overextension of monosyllable words” hypothesis [4]. The result suggested that the hypothesis may hold under some cases, but not for all of the monosyllable words. In summary, the result indicated that more than 80% of the participants agreed that there were word boundaries before and after the conjunctions, perhaps because the conjunctions are used to connect words, phrases or clauses in Chinese. Thus, consistent with the rules of the *CCLWSSIP*, most Chinese readers agreed that the conjunction could be considered a single word unit.

#### Prepositions

**Figure 7:**
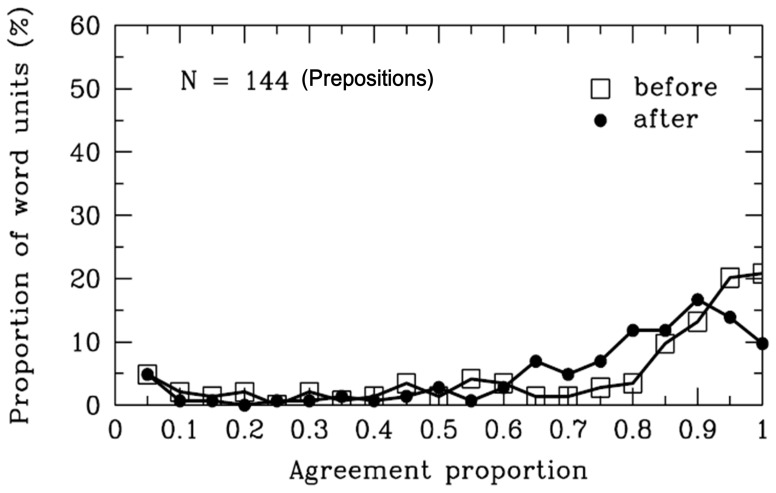
Prepositions. Frequency distribution of agreement proportions for word boundaries before (open squares) and after (filled circles) the prepositions. The y-axis represents the proportion of SWU. The number of SWU is also represented in the figure.

As is evident in Table 1 and Figure 7, the agreement proportions for word boundaries before [*M* = .74, *SD* = .29, *t* (143) = 9.95, *p*<.001] and after [*M* = .74, *SD* = .24, *t* (143) = 11.98, *p*<.001] the prepositions were significantly higher than the value .50. There were 27 different prepositions segmented in the materials, and 97% of the 144 prepositions were 1-character words. Like the conjunctions, participants’ segmentation rules for the prepositions did not support the “overextension of monosyllable words” hypothesis [4].

Additionally, the results suggested several possible rules of word segmentation be used by ordinary Chinese readers. In some special cases in our materials, participants were uncertain whether the preposition could be considered a single word unit. For example, some prepositions can be put after verbs to function as a complement, such as “
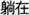
” (lie down). Most readers tended to combine the preposition “
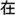
” (down) with the verb “
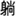
” (lie) together as one word. However, one limitation of our materials was that the percentage of these cases was low. In this case, it might have been difficult for Chinese readers to decide whether there are word boundaries before the prepositions. Nevertheless, in most cases of the materials, more than 70% of participants agreed that the prepositions could be considered single word units. It appeared to be consistent with the rules of the *CCLWSSIP*.

### Content Words

#### Adjectives

**Figure 8:**
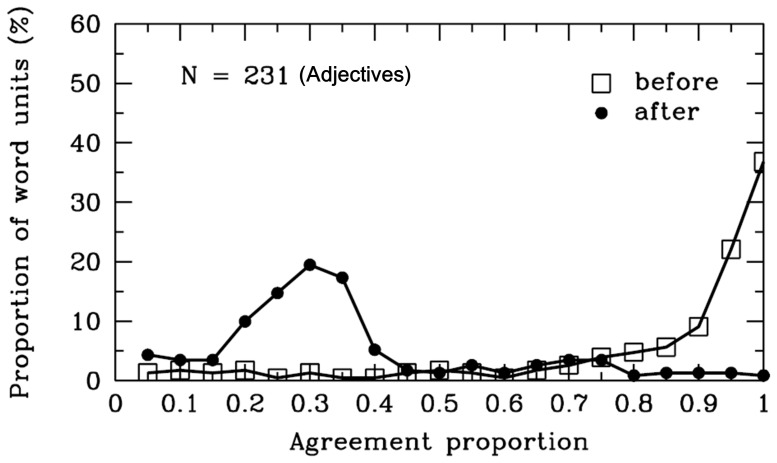
Adjectives. Frequency distribution of agreement proportions for word boundaries before (open squares) and after (filled circles) the adjectives. The y-axis represents the proportion of SWU. The number of SWU is also represented in the figure.

**Figure 9:**
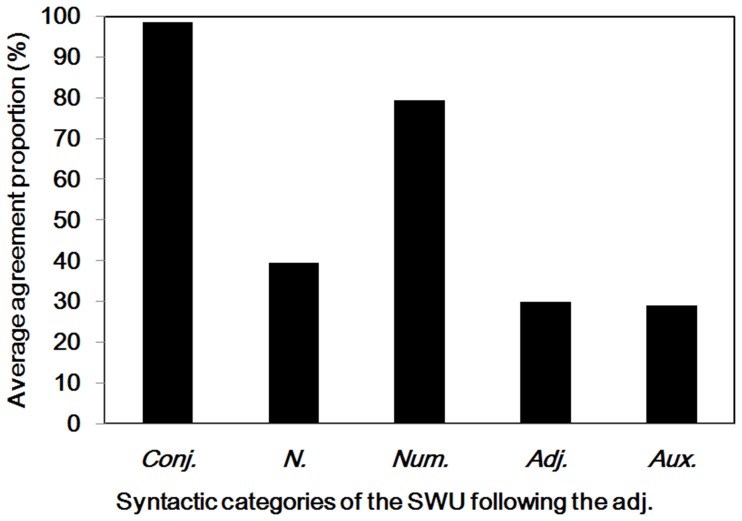
Adjectives and subsequent words. Average agreement proportions for word boundaries between the adjectives and different subsequent syntactic categories of SWU. Conj. = conjunctions; N. = nouns; Num. = numerals; Adj. = adjectives; Aux. = auxiliary words.

As is evident in Table 1 and Figure 8, the agreement proportion for word boundaries before the adjectives (*M* = .83, *SD* = .24) was significantly higher than .50 [*t* (230) = 20.48, *p*<.001]. However, the agreement proportion after the adjectives (*M* = .34, *SD* = .21), was significantly lower than .50, *t* (230) = −11.53, *p*<.001. There were 126 different adjectives segmented in the materials, and the word length varied from one to four characters. To clarify why participants were uncertain whether there were word boundaries after the adjectives, we analyzed the syntactic categories of SWU after the adjectives. There were 231 SWU of the adjectives in the materials. A total of 59% of the adjectives were followed by the auxiliary words, and 33% of the adjectives were followed by the nouns. Among these items, the agreement proportions for word boundaries between the adjectives and the auxiliary words or the nouns were lower than .40, significantly lower than .50 (*ps* <.001). Interestingly, 65 adjective units and the subsequent nouns can form modifier-core phrases, in which the agreement proportion for word boundaries between the adjectives and the subsequent nouns was .46 (*SD* = .26). Specifically, the agreement proportions between the adjectives and the conjunctions or numerals were higher than .79 (see Figure 9).

In summary, the results suggested that most Chinese readers agreed that there were word boundaries before the SWU of adjectives. The participants were uncertain whether there were word boundaries between the adjectives and the subsequent words when the syntactic categories of subsequent words were auxiliary words, nouns, and other adjectives. In these cases, Chinese readers could not follow the rules of the *CCLWSSIP*, which consider each adjective or adjacent component as a single word. Chinese readers could follow the rules of the *CCLWSSIP* when the adjective words were followed by the conjunctions or numerals. It appears that the word segmentation pattern depends on the syntactic relationship between the consecutive words.

#### Nouns

**Figure 10:**
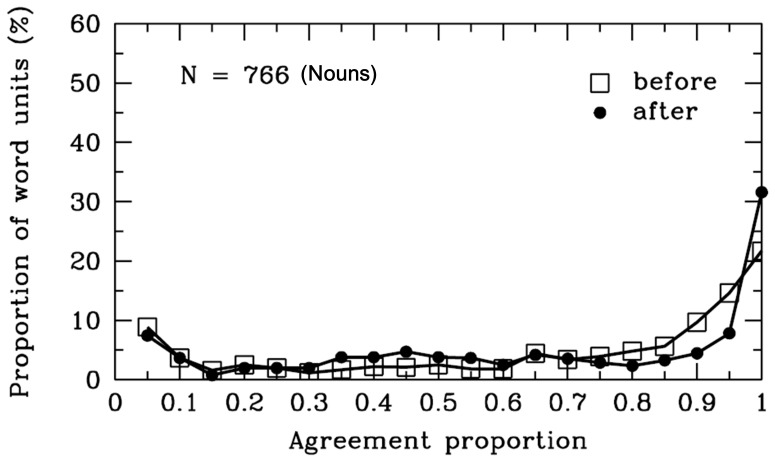
Nouns. Frequency distribution of agreement proportions for word boundaries before (open squares) and after (filled circles) the nouns units. The y-axis represents the proportion of SWU. The number of SWU is also represented in the figure.

The agreement proportion for word boundaries before [*M* = .67, *SD* = .33, *t* (765) = 14.41, *p*<.001] and after [*M* = .66, *SD* = .33, *t* (765) = 13.26, *p*<.001] the nouns was significantly higher than .50 (see Table 1). Actually, it might have been challenging for the participants to decide whether there were word boundaries before or after the nouns (see Figure 10). Nouns are words that can be used to name animals, persons, things, places, abstract ideas, etc. There were 349 different nouns segmented and 766 SWU of the nouns in the materials. A total of 17% of the 766 SWU contained 1 character. The agreement proportion for word boundaries before [*M* = .21, *SD* = .30, *t* (127) = −10.88, *p*<.001] the 1-character nouns was significantly lower than .50; this proportion after the 1-character nouns (*M* = .66, *SD* = .38) was significantly higher than .50, *t* (127) = 4.92, *p*<.001. For the 2-character nouns (77% of nouns), both the agreement proportion for word boundaries before [*M* = .76, *SD* = .25, *t* (592) = 24.87, *p*<.001] and after [*M* = .65, *SD* = .33, *t* (592) = 11.38, *p*<.001] the nouns were significantly higher than .50. This finding indicated that word length may have an effect on word segmentation. The results appeared to support the “overextension of monosyllable words” hypothesis [4].

**Figure 11:**
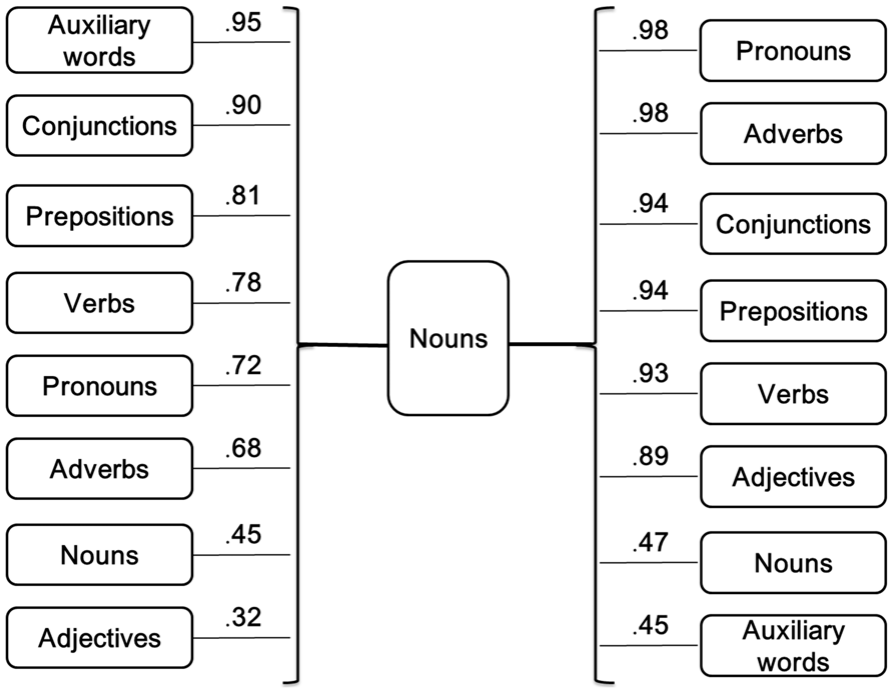
Nouns and adjacent words. Average agreement proportions for word boundaries between the nouns and different adjacent syntactic categories of SWU. The left values of the figure show the average agreement proportion between the preceding words and the nouns, and the right values of the figure show the average agreement proportion between the nouns and the subsequent words.

To clarify the role of nouns in word segmentation, we analyzed the agreement proportion between the nouns and their adjacent words in detail. We analyzed the syntactic categories of SWU before and after the nouns (see Figure 11). The results showed that the participants were uncertain about the word boundaries of the nouns in some cases. A total of 6% of the nouns were preceded by adjectives, and the average agreement proportion between the adjectives and the nouns was .32; 14% of nouns were followed by auxiliary words, and the average agreement proportion between the nouns and the auxiliary words was .45. In addition, more than 20% of the nouns were preceded or followed by other nouns. The average agreement proportion between these consecutive nouns was no more than .50. The participants might be uncertain how to segment the consecutive nouns, perhaps because these consecutive nouns might express global meanings or share a range of linguistic properties. For instance, the phrase “
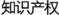
” (i.e., intellectual property) could be considered as a whole by ordinary Chinese readers, since the word “
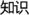
” (i.e., intellectual) and “
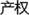
” (i.e., property) may share the same syntactic constitute in a sentence. These cases are inconsistent with the rules of the *CCLWSSIP*.

However, when the verbs, adverbs, conjunctions, pronouns or prepositions were adjacent to the nouns (see Figure 11), most participants agreed that the nouns could be considered single word units. Perhaps these adjacent words and nouns express different meanings in the sentence reading. These cases are consistent with the rules of the *CCLWSSIP*. It is possible that the discrepancies for decisions whether there are word boundaries between the strings of characters may depend on the syntactic relationship between consecutive words.

#### Numerals and quantifiers

**Figure 12:**
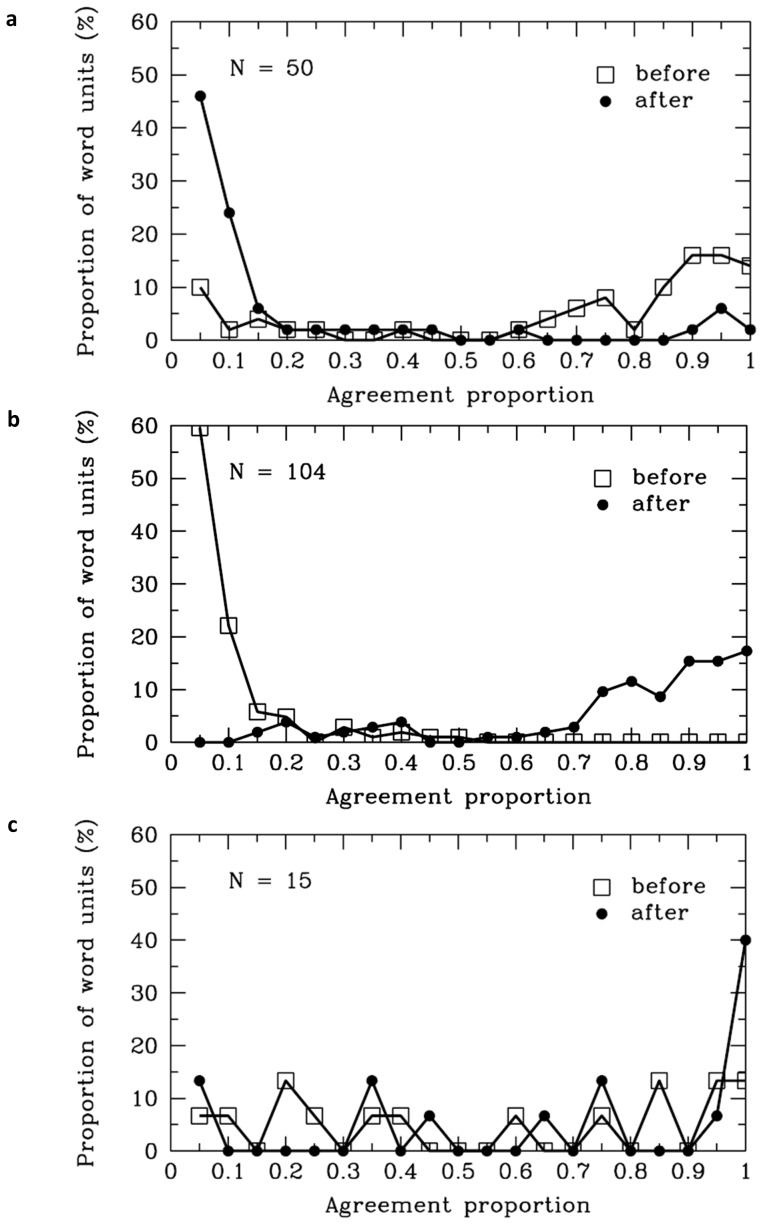
Numerals and Quantifiers. Frequency distribution of agreement proportions for word boundaries before (open squares) and after (filled circles) the numerals (a), the quantifiers (b), the numeral & quantifiers (c). The y-axis represents the proportion of SWU. The number of SWU was also present in each panel of the figure.

There were 50 SWU of numerals, 104 SWU of quantifiers, and 15 SWU of numeral and quantifier units in the materials based on the participants’ delimitation of word boundaries. The agreement proportion for word boundaries after the quantifiers (*M* = .76, *SD* = .24) was significantly higher than .50, *t* (103) = 11.21, *p*<.001; and the agreement proportion before the quantifier word units (*M* = .08, *SD* = .09) was significantly lower than .50, *t* (103) = −45.76, *p*<.001 (see Table 1 and Figure 12b). The results indicated that most participants agreed that there were word boundaries after the quantifiers, but they disagreed as to whether there were word boundaries before the quantifiers. Additionally, the results showed that participants tended to agree that there were word boundaries before the numerals [*M* = .69, *SD* = .33, *t* (49) = 4.12, *p*<.001], but they denied that there were word boundaries after the numerals [*M* = .18, *SD* = .28, *t* (49) = −8.04, *p*<.001] (see Figure 12a). Additionally, 78% of the numerals and 99% of the quantifiers were 1-character words. The segmentation results showed that most participants tended to combine the numeral and the quantifier to form a single word. This finding appeared to support the “overextension of monosyllable words” hypothesis. Specifically, 15 SWU of numerals and quantifiers were considered to be single word units by all the participants, though the agreement proportions for the word boundaries before [*M* = .54, *SD* = .35, *t* (14) = .47, *p* = .65] and after [*M* = .68, *SD* = .35, *t* (14) = 1.95, *p* = .07] the numeral & quantifier units were not significantly different from .50 (see Figure 12c).

In summary, the results suggested that most Chinese readers disagreed that there were word boundaries between the numeral and the quantifiers, a conclusion that was inconsistent with the rules of the *CCLWSSIP*. According to the rules of the *CCLWSSIP*, the numeral and quantifiers should be considered as distinct words. One possible reason for the inconsistent findings may be resulted from the functions of numerals and quantifiers. Numerals are used to describe how many or how much, and the quantifiers are often set between the numerals and nouns in Chinese, for example 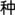 (i.e., type of), 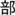 (i.e., part of), and 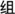 (i.e., group of). In most cases, numerals and quantifiers are used together to precede or modify nouns. Additionally, the numerals and the quantifiers share a range of linguistic (e.g., syntactic, semantic, and pragmatic) properties [31]. Thus, unlike the rules of the *CCLWSSIP*, most Chinese readers tended to combine the numeral and the quantifier as a single word unit.

#### Pronouns

**Figure 13:**
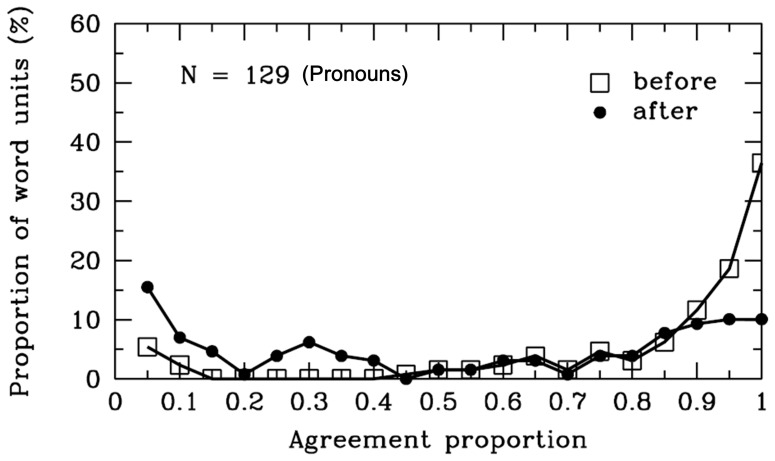
Pronouns. Frequency distribution of agreement proportions for word boundaries before (open squares) and after (filled circles) the pronouns. The y-axis represents the proportion of SWU. The number of SWU is also represented in the figure.

**Figure 14:**
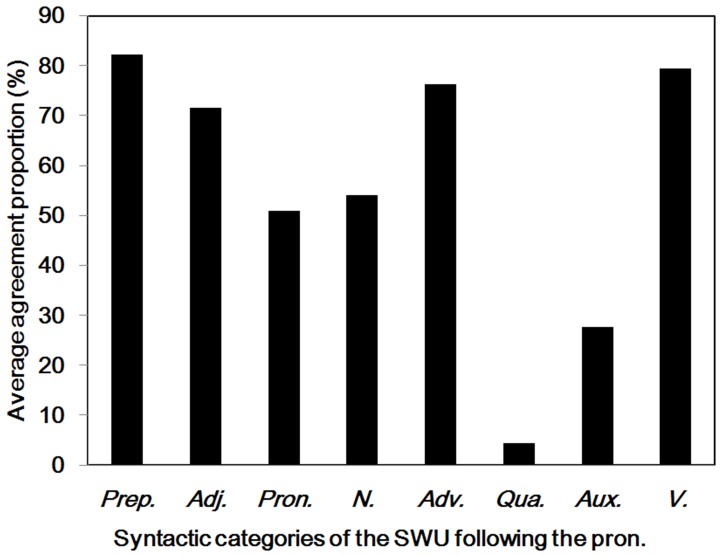
Pronouns and subsequent words. Average agreement proportions for word boundaries between the pronouns and different subsequent syntactic categories of SWU. Prep. = prepositions; Adj. = adjectives; Pron. = pronouns; N. = nouns; Adv. = adverbs; Qua. = quantifiers; Aux. = auxiliary words; V. = verbs.

Pronouns can replace a noun or another pronoun to make the sentences less repetitive. The agreement proportion for word boundaries before the pronouns (*M* = .82, *SD* = .26) was significantly higher than .50, *t* (128) = 13.81, *p*<.001; however, the agreement proportion for word boundaries after the pronouns (*M* = .52, *SD* = .37) did not significantly differ from .50, *t* (128) = .72, *p* = .48 (see Table 1 and Figure 13). There were 32 different pronouns segmented in the materials, and the word length varied from one to three characters. To clarify why participants were uncertain whether there were word boundaries after the pronouns, we analyzed the syntactic categories of SWU after the pronouns (see Figure 14). A total of 18% and 17% of the pronouns were followed by the auxiliary words and quantifiers, respectively, and the agreement proportions for word boundaries between the pronouns and the auxiliary words or the quantifiers were less than .30, significantly lower than .50 (*ps* <.001). In contrast, when the pronouns were followed by the prepositions, verbs, adverbs, and adjectives, the agreement proportion for word boundaries between the pronouns and these types of syntactic words was above .70, which was significantly higher than .50 (*ps* <.001).

In summary, the results showed that most Chinese readers agreed that there were word boundaries before the pronouns, but the participants were uncertain whether there were word boundaries after the pronouns. Their decision may depend on what the subsequent word was. When the pronouns were followed by the auxiliary words or quantifiers, most Chinese readers tended to combine the pronouns with the subsequent words to form single word units. These cases were inconsistent with the rules of the *CCLWSSIP*. However, when the pronouns were followed by the verbs, adverbs, prepositions or adjectives, most Chinese readers tended to consider the pronouns as single word units. The cases were consistent with the rules of the *CCLWSSIP*.

#### Verbs

**Figure 15:**
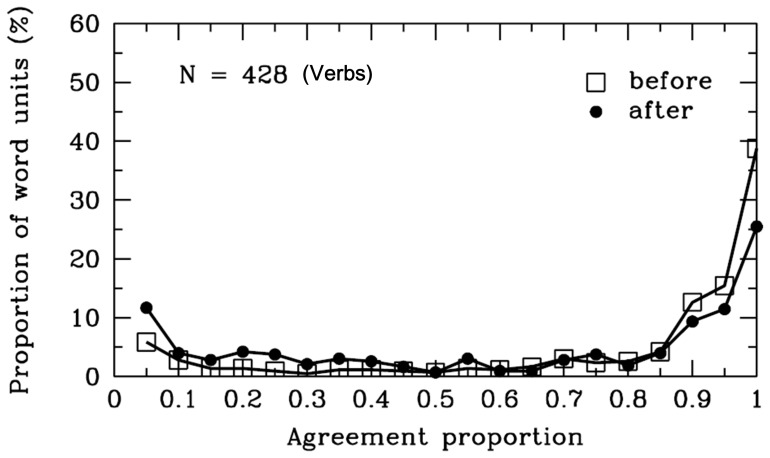
Verbs. Frequency distribution of agreement proportions for word boundaries before (open squares) and after (filled circles) the verbs. The y-axis represents the proportion of SWU. The number of SWU is also represented in the figure.

**Figure 16:**
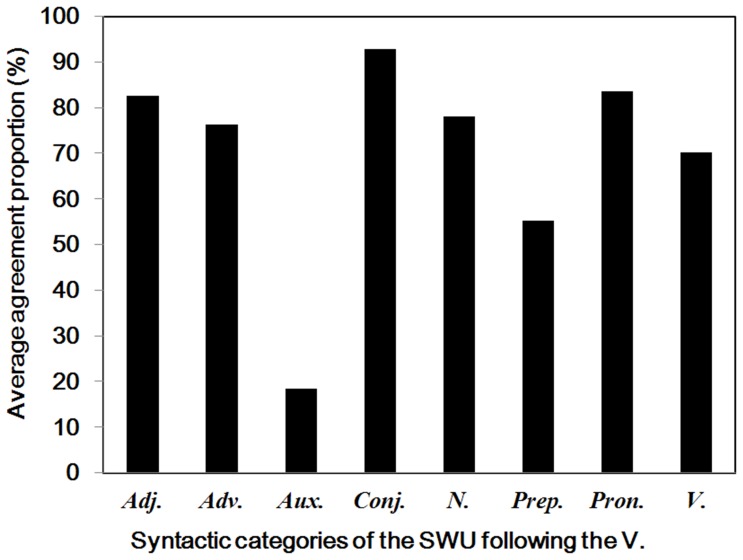
Verbs and subsequent words. Average agreement proportions for word boundaries between the verbs and different subsequent syntactic categories of SWU. Adj. = adjectives; Adv. = adverbs; Aux. = auxiliary words; Conj. = conjunctions; N. = nouns; Prep. = prepositions; Pron. = pronouns; V = verbs.

As shown in Table 1 and Figure 15, the agreement proportions for word boundaries before [*M* = .78, *SD* = .30, *t* (427) = 19.27, *p*<.001] and after [*M* = .62, *SD* = .37, *t* (427) = 6.98, *p*<.001] the verbs were significantly higher than .50. The results indicated that most participants agreed that there were word boundaries before the verbs. However, participants may have been uncertain whether there were word boundaries after the verbs in some cases. The verbs are words that convey actions, events or states of being. There were 174 different verbs in the materials, and the word length varied from one to three characters. To clarify the inconclusive cases, we analyzed the syntactic categories of SWU after the verbs (see Figure 16). The results showed that 19% of the verbs were followed by auxiliary words, and the agreement proportions for word boundaries between the verbs and the auxiliary words was .18 (*SD* = .14), which was significantly lower than .50 (*p*<.001). In addition, 9% of the verbs were followed by prepositions, and the agreement proportion between the verbs and the prepositions was .55. The results showed that most participants were uncertain whether there were word boundaries after the verbs which were followed by the auxiliary words or prepositions. Interestingly, the cases are consistent with what we have reported above. Most participants denied that there were word boundaries before the auxiliary words. Additionally, some participants tended to combine the verb and the preposition to form a verb-complement structure which is an important grammatical feature in Chinese. These cases are inconsistent with the rules of the *CCLWSSIP*. Specifically, 62% of the verbs were followed by the adjectives, adverbs, conjunctions, nouns, and pronouns; and the agreement proportions between the verbs and these words were higher than .70. The results showed that most participants agreed that there were word boundaries after the verbs that were followed by the adjectives, adverbs, conjunctions, nouns, pronouns, or other verbs. The cases were consistent with the rules of the *CCLWSSIP*.

### The Notion of Words by Ordinary Chinese Readers

As described above, one hundred and forty two participants were invited to write down what they thought of the concept of words. One participant skipped this part, thus there were 141 responses in total. Word is defined as the smallest independent and meaningful unit of language according to Chinese linguistics [31]. The definition of Chinese words contains four features: smallest, independent, meaningful, and unit. Among the 141 responses, 87% reported that words must have meanings or senses that convey some information (e.g., meanings, ideas, events, actions, etc.), 18% reported that the word was a unit of language, 14% reported that a word is independent, and 9% reported that a word was the minimum unit or component in reading. Interestingly, only 1.42% of the responses completely matched the definition of the words. Additionally, 53% (i.e., 75) of the total responses mentioned that Chinese words were composed of several characters. Among the 75 responses, 23% reported that Chinese words contained two or more characters, and 13% reported that words were also phrases or clauses.

When we counted the number of word units segmented in the materials, we found that the number of word units segmented based on the *CCLWSSIP* was significantly larger than the number of word units segmented by each participant (*p*<.001). A possible reason for this finding is that few participants believed that a Chinese word must be minimum unit. Chinese readers might have combined several characters or words to form a single “word” as an informational unit, and the number of informational units should be less than the number of words based on the rules of the *CCLWSSIP*. It is possible that fewer informational units may reduce the processing load and improve the processing efficiency of the readers. Another possible reason is that most Chinese readers believed that Chinese words must have substantial meanings. The results showed that most Chinese readers tended to combine some function words (e.g., auxiliary words) with content words to form single word units, perhaps because these function words have little substantial meaning. Thus, the number of word units segmented by the *CCLWSSIP* was higher than that by the participants.

## General Discussion

We conducted a preliminary study to examine whether Chinese readers follow the national standard rules of word segmentation based on the *CCLWSSIP* when they were asked to segment sentences into individual words. The results showed that Chinese readers did not entirely follow the segmentation rules of the *CCLWSSIP*, and their segmentation processing were strongly influenced by the syntactic categories of consecutive words. It is possible that the concept of words for Chinese readers is different from the word definition by linguistics and the *CCLWSSIP*. Additionally, our results tested the “overextension of monosyllable words” hypothesis and showed that it was right in some occasions and might need to be corrected to some degree. Furthermore, the findings regarding word segmentation rules by Chinese readers may have implications for information processing and computational models of Chinese reading.

### Comparison of the Word Segmentation Rules Used by Chinese Readers and the *CCLWSSIP*

Chinese readers’ spontaneous word segmentation processing may be complex. According to the rules of the *CCLWSSIP*, each syntactic category could be considered as a single word unit. However, the present study showed that Chinese reader did not totally follow the word segmentation rules of the *CCLWSSIP*. As showed in the Results section, based on the syntactic categories of consecutive words, the data were sorted in describing the consistency and inconsistency between the word segmentation rules used by Chinese readers and the *CCLWSSIP*. In summary, three kinds of situations were detected in the present study.

In the first situation, most Chinese readers reached a consensus on word boundaries that were consistent with the rules of the *CCLWSSIP*. Our results showed that Chinese readers agreed that there were word boundaries before and after the conjunctions and prepositions in most cases. As noted above, the function of the conjunctions is to connect words, phrases or clauses that are coordinate components in the sentence. A preposition usually links nouns, pronouns or phrases to other words in a sentence. In these cases, the conjunctions and prepositions may be regarded as independent units for ordinary Chinese readers. Moreover, the results showed that Chinese readers followed the rules of the *CCLWSSIP* in other cases: a) when the adjectives were followed by the conjunctions or numerals; b) when the nouns were adjacent to the verbs, adverbs, conjunctions, pronouns or prepositions; c) when the pronouns were followed by the verbs, adverbs, prepositions or adjectives; d) when the verbs were followed by adjectives, adverbs, nouns, pronouns, or other verbs. In these cases, most participants considered the adjectives, nouns, pronouns, and verbs to be single word units as the *CCLWSSIP*.

Second, most Chinese readers in our study reached a consensus on the word boundaries, but their segmentation processing did not follow the rules of the *CCLWSSIP*. A typical case pertained to the auxiliary words. When the auxiliary words were preceded by the adjectives, adverbs, nouns, pronouns or verbs, most Chinese readers considered the combination of the auxiliary words and the adjacent words to be single word units. In addition, most Chinese readers agreed that the numerals and quantifiers should be combined together as single word units in the present study. Furthermore, the results showed that Chinese readers agreed that there were no word boundaries between the consecutive words in the following cases: a) when the nouns were preceded by the adjectives; b) when the pronouns were followed by the quantifiers; c) when the verb and the preposition formed a verb-complement structure. Thus, although Chinese readers reached a consensus in these cases, they did not follow the rules of the *CCLWSSIP*.

In the third situation, Chinese readers experienced difficulty in agreeing on the word boundaries for the same text. The results showed that Chinese readers were uncertain whether word boundaries should be inserted in the following cases: a) when the adverbs were followed by adjectives, prepositions, verbs or other adverbs; b) when the adjectives and the subsequent nouns could form modifier-core phrases; c) when consecutive nouns were present. Under these cases, the participants’ average agreement proportions for word boundaries were not significantly different from the chance level.

In summary, the results showed that Chinese readers did not follow the word segmentation rules of the *CCLWSSIP*, and their spontaneous word segmentation processing was strongly influenced by the syntactic categories of consecutive words.

### “Overextension of Monosyllable Words” Hypothesis

Peng and Chen (2004) proposed the “overextension of monosyllable words” hypothesis, which may be an important causal factor for word segmentation inconsistency. This hypothesis reports that most Chinese readers tend to combine monosyllables with adjacent disyllables to form a “word”. In nature, it indicates that word length have an effect on Chinese readers’ word segmentation processing. Our results also provided evidence to support this hypothesis. For instance, in our materials, most of the auxiliary words, numerals, quantifiers and some of the nouns were 1-character words, and we indeed found that readers tended to combine these words with other adjacent characters to form single word units. Additionally, approximately half of the participants mentioned that Chinese words were composed of several characters. These results suggested that the “overextension of monosyllable words” phenomenon was common when Chinese readers were asked to identify words during Chinese sentences processing.

However, our results suggested that the hypothesis may be reasonable for some but not all of the monosyllable words. Interestingly, most Chinese readers tended not to combine monosyllable conjunction or preposition with the adjacent characters to form a single word. Instead, conjunctions and prepositions were regarded as independent units by Chinese readers. This finding suggested that “overextension of monosyllable words” hypothesis could not be applied to all kinds of monosyllables. In summary, if monosyllable words and the adjacent characters could form strong meaningful word units, Chinese readers’ word segmentation processing tended to support the “overextension of monosyllable words” hypothesis. Otherwise, they may not support the hypothesis.

### Alternative Explanations of Chinese Readers’ Word Segmentation Rules

Although our results showed that Chinese readers could reach a consensus on the word boundaries sometimes, they did not follow the segmentation rules by the *CCLWSSIP* in many cases. An important question is why Chinese readers did not follow the national rules of word segmentation based on the *CCLWSSIP*. There may be several reasons.

First, word semantic substance may have an effect on readers’ segmentation processing. More than 80% of the participants reported that Chinese words must have several meanings or senses which convey information like ideas, events, actions, etc. Content words, such as the nouns, verbs, adjectives, etc., have stable lexical meanings, so that they were easy to be marked the boundaries in a sentence by the participants. In contrast, the function words usually have minimal semantic substance and are typically used to indicate a syntactic function [2], [28]–[30], thus they were difficult for the participants to mark the word boundaries. Moreover, most function words are less complicated and occur with higher frequency than content words, such that they are more likely than content words to be skipped over, as eye movement studies have indicated [33]. Rayner et al. (2007) also found that disagreements concerning word boundaries primarily occurred for function words. Thus, word semantic substance may have an effect on Chinese readers’ word segmentation, and the participants tended to combine function words with content words to form single word units.

However, it is not always difficult for Chinese readers to identify word boundaries of function words, such as the conjunctions and prepositions. Meanwhile, it is not always easy to mark word boundaries of content words (e.g., when consecutive nouns were present, or when the numerals and quantifiers were present together). These cases suggested, besides semantic substance, that the relationship among consecutive words may also influence Chinese readers’ word segmentation processing. Our results showed that the conjunctions and prepositions were often used to connect content words in the materials, and the relationship among these consecutive words appeared to be independent. Consequently, the conjunctions and prepositions were likely to be considered as single word units by the participants, respectively. Nevertheless, these consecutive content words may express global meanings or share a range of linguistic properties, and the relationship between these words were close, so that the participants marked them together as single word units. Thus, the intensity of relationship between a string of characters may influence Chinese readers’ word segmentation processing, which did not follow the rules of the *CCLWSSIP*.

Third, the characteristics of Chinese reading may play a critical role in word boundary inconsistencies. First, there are no explicit markers that specify grammatical categories in consecutively written texts [1], [2]. As a result, a word in Chinese text does not have salient or distinctive characteristics compared to other structural units of the language, such as morpheme and phrases. Second, there are no word length cues for words in sentences. A Chinese word may be formed by various numbers of characters, ranging from 1 character to 15 characters, as described in the *Chinese Lexicon* (2003). Third, most individual characters have multiple meanings when combined with different characters [1], such that word boundaries may be determined by both lexical knowledge and sentence context information [2], [8], [27]. Thus, it may be difficult for Chinese readers to determine the word boundaries during reading.

Fourth, Chinese readers’ vague concept of words may have an influence on the inconsistent word boundaries. As noted above, most participants considered Chinese words should have “meanings” or refer to “one thing/idea”. However, the boundary of what a participant believed to be one thing or one idea might have varied, according to whichever informational unit the participant focused on at a time. Furthermore, half of the participants also thought Chinese words might contain several characters. Nevertheless, according to the *CCLWSSIP*, each syntactic category is considered as a single word unit regardless of the meaning or number of characters. Thus, Chinese readers’ vague concept of words may have an effect on the word segmentation. It is possible that Chinese readers could follow the national rules if they were informed the definition of a word and the rules of the *CCLWSSIP* in advance.

Finally, a possible reason that Chinese readers did not follow the segmentation rules of the *CCLWSSIP* is that they may adopt a strategy by using large chunks to decrease cognitive load in order to improve comprehension. A larger number of previous studies have demonstrated the importance of grouping or organizing the input sequence into units or chunks (e.g., [34], [35]). The results showed that the number of word units segmented by each participant was less than that segmented based on the *CCLWSSIP*. Additionally, few Chinese readers mentioned that word units were the smallest or independent units. Actually, the “overextension of monosyllable words” phenomenon essentially reflected that Chinese readers tended to combine small units to form large chunks during Chinese reading. This large chunks may decrease the number of information units and working memory load, thereby improving their reading comprehension. In the present study, readers’ purpose may be primarily to understand sentences effectively, and therefore they segmented the sentences into words according to their own reading habits in such a way that it was unnecessary for them to follow the national guidelines of the *CCLWSSIP*.

To some extent, the goal of the segmentation rules by both Chinese readers and the *CCLWSSIP* may be similar: to understand sentence clearly and process linguistic information effectively. From the perspectives of psycholinguistics, the potential word segmentation strategies of Chinese readers may assist practitioners of computational linguistics who work on information processing. Until now, there are some inevitable errors of automatic word segmentation in computational linguistics. For instance, the automatic techniques could not figure out the segmentation problems on ambiguous phrases like “
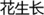
” which may be segmented as “
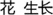
” (means “flower grows”) or “
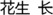
” (means “peanut grows”). However, to resolve these segmentation ambiguous problems, Chinese readers can use the information of sentence context. Our results indicated that syntactic categories of consecutive words also provide critical cues for Chinese readers’ word segmentation processing. Meanwhile, Chinese readers tended to use larger chunks to decrease cognitive load in sentence comprehension relative to the rules of the *CCLWSSIP*. In summary, the strategies adopted by Chinese readers using sentence context and large chunks may have implications for automatic word segmentation and effective information processing.

### Implications of Chinese Readers’ Word Segmentation Rules for Computational Models

Several researchers have suggested that understanding what information readers use to identify word boundaries is necessary for developing computational models of sentence reading in non-alphabetic languages [9], [36], [37]. However, most theoretical developments have been proposed based on alphabetic languages such as English, while relatively little work has been conducted on non-alphabetic languages such as Chinese. The present study obtained abundant data on ordinary Chinese readers’ spontaneous word segmentation processing in reading, which may have implications for the developments of word recognition and eye movement control models in Chinese.

Most current theoretical models of Chinese word identification have been limited to single words without sentence context [8], [9], [11]. These models do not include any mechanism for the word segmentation inconsistency in Chinese reading, such as the Lexical Constituency Model which proposed that the words are represented across orthography, phonology, and semantics [11]. Recently, Li et al. (2009) proposed a word segmentation and word recognition model which assumes that Chinese word recognition involves multiple levels of processing consisting of a visual perception level, a character recognition level, and a word segmentation and recognition level. The model also assumes that the segmentation and identification of words are not distinguishable, and that the two processes are interactive involving top-down and bottom-up factors. Although this model enhances the understanding of word segmentation, it does not consider the influence of context information or word syntactic categories that may play an important role in word segmentation during Chinese reading.

Our results concerning Chinese readers’ word segmentation rules may provide new information for the architecture of word recognition models. Although word recognition models assume that Chinese word can be represented across different levels, these models have largely ignored the issue of how sentence context or word boundaries between consecutive words affects word recognition. Several prior studies have demonstrated that the identification of word boundaries must be vital for successful word identification [8], [9], [36], [38]. The present study showed that Chinese readers’ word segmentation processing may be influenced by semantic substance, relationship between consecutive words, strategy like using large chunks, and etc. Thus, following the model proposed by Li et al. (2009), our results suggested that Chinese word recognition may involve multiple sub-levels of processing including visual perception, character recognition, word segmentation and sentence context level. The present study may have made considerable progress in developing a deeper understanding of some of the components of the word recognition in sentence reading.

Additionally, our findings may have implications for the eye movement control models of Chinese, which have been reported by very little literature until now. Rayner et al. (2007) first extended one of the most influential models of eye movement control in alphabetic languages (i.e., E–Z Reader) to Chinese reading. During the processing of computational modeling, they assumed that readers agreed on the word boundaries in Chinese reading. Although there are some differences between Chinese and English (e.g., interword spaces), the model simulated the eye movement of Chinese readers, which were similar to those in English reading as indexed by fixation duration and fixation probability. Finally, the study suggested that the basic architecture of the E–Z Reader model could account for the eye movement control of Chinese readers.

However, it is vague that whether some basic assumptions of the E–Z Reader model in English could generalize for that in Chinese. For instance, one basic assumption of the E–Z Reader is a serial word processing in reading in which word n+1 is processed only after the lexical processing of word n is completed (i.e., only one word could be processed at a time) [39]–[42]. Nevertheless, it is unclear how many words or characters may be processed at a time in Chinese. Relative to English words, Chinese words contain greater information density and word length in Chinese is generally shorter. Our results showed that, in many cases, it is hard for Chinese readers to identify one word from a string of characters. Meanwhile, in order to improve reading efficiency, Chinese readers tended to use the lexical knowledge and sentence context to segment sentence into larger information units relative to the rules by the *CCLWSSIP*. Thus, the basic assumption of eye movement control models of alphabetic languages may not directly generalize to that of Chinese. We propose that Chinese readers may process an information unit rather than a word at a time. In summary, the present results of word segmentation rules by Chinese readers should be taken into account in the next generation of computational models of word recognition and eye movement control during Chinese reading.

### Conclusions

The present study examined how Chinese readers segment the sentence into individual words given the paucity of visual word-boundary cues in Chinese reading. Chinese readers’ word segmentation processing tended to be flexible, and they did not follow the rules of word segmentation based on the *CCLWSSIP*. In the past 20 years, education in China has changed significantly. Nevertheless, we found that most Chinese readers were still vague to the notion of the word, a finding that is consistent with that of Hoosain (1992). The current results may stimulate more studies to explore the mechanism of Chinese readers’ word segmentation processing. However, there are several limitations in the present study. First, the materials used in the current study did not contain all of the syntactic categories with equal weight. If more elaborated materials were selected, the findings might be improved. Second, the study used self-reporting method to explore word segmentation during Chinese reading. This method is sometimes unreliable; thus, some of the results should be treated with caution and tested in further studies. We believe that the findings presented in the current study will enhance the understanding of word segmentation mechanisms in Chinese even with some limitations.

## Supporting Information

Appendix S1 **An illustration of the coding of the agreement proportion for word boundaries.**
(DOCX)
