# Supplementary material for: Do Chinese Readers Follow the National Standard Rules for Word Segmentation during Reading?
Source: PLoS One. 2013 Feb 8;8(2):e55440. doi: 10.1371/journal.pone.0055440 (PMC3568123; doi:10.1371/journal.pone.0055440)
Supplement: Appendix S1 — An illustration of the coding of the agreement proportion for word boundaries. (DOCX) [file pone.0055440.s001.docx]

# **Appendix S1**

An illustration of the coding of the agreement proportion for word boundaries.

A simple sentence for illustration (not used in the experiment):

祝愿您快乐每一天 (i.e., I wish you happiness every day)

There are 8 characters and 7 word boundary candidates in the sample sentence. The participants were asked to segment the normal Chinese sentences into individual words using slashes (“/”), such as 祝愿/您/快乐/每一天. This process can be further coded into a string of “0”s and “1”s, in which 0 indicates “not followed by a boundary”, and 1 indicates “followed by a boundary”. We can calculate the number of “/” after each character based on the coding. We assumed that 50 participants were asked to segment the simple sentence. The number of “/” after each character was assumed to be: 祝(0)愿(40)您(50)快(0)乐(48)每(18)一(3)天. Then, the Chinese words “祝愿”, “您”, “快乐”, “每”, “一”, and “天” were considered to be 6 segmented word units (SWU). Finally, the agreement proportions after each SWU were 祝愿(.80)您(1.00)快乐(.96)每(.36)一(.06)天. For instance, for the SWU “您” (i.e., you), the agreement proportion before the SWU “您” was .80, and the agreement proportion after the SWU 您was 1.00.
